# A Novel Case of Hypoparathyroidism Secondary to SARS-CoV-2 Infection

**DOI:** 10.7759/cureus.10097

**Published:** 2020-08-28

**Authors:** Sherif Elkattawy, Ramez Alyacoub, Sarah Ayad, Manthan Pandya, Ari Eckman

**Affiliations:** 1 Internal Medicine, Rutgers-New Jersey Medical School/Trinitas Regional Medical Center, Elizabeth, USA; 2 Internal Medicine-Endocrinology, Robert Wood Johnson University Hospital, New Brunswick, USA; 3 Internal Medicine-Endocrinology, Rutgers-New Jersey Medical School/Trinitas Regional Medical Center, Elizabeth, USA

**Keywords:** hypoparathyroidism, hyperphosphatemia

## Abstract

Hypoparathyroidism is usually caused by postsurgical or autoimmune damage to the parathyroid gland. We present the case of a 46-year-old Hispanic male with no significant past medical history who was admitted to the hospital with hypoxic respiratory failure due to coronavirus disease 2019 (COVID-19) infection and had a prolonged hospital course. He was incidentally found to have hyperphosphatemia and low parathyroid hormone (PTH) levels. During the second month of hospitalization, his phosphorus levels rose to 6.9 mg/dL (normal range: 2.4-4.7 mg/dl). His PTH levels were found to be at 8 pg/mL. Vitamin D levels obtained were also low (7 ng/dL), phosphorus was at 5.8 mg/dL with albumin of 2.9 g/dL, and calcium level was normal at 9.2 mg/dl. Parathyroid hormone-related peptide (PTHrP) level was low at 10. Malignancy and genetic causes were ruled out. The patient was started on 50,000 units of ergocalciferol once a week. He was also started on calcium acetate 1,334 mg three times a day for hyperphosphatemia. Phosphorus levels remained elevated, and sevelamer was added on discharge after he was weaned off oxygen and cleared by physical therapy. No explanation for persistent hyperphosphatemia and hypoparathyroidism was found. To date, there have been some reports linking severe acute respiratory syndrome coronavirus 2 (SARS-CoV-2) to widespread tissue injury; however, there have been no reports so far on the effect of the parathyroid gland. Further studies are necessary to elaborate and to confirm the causative relationship between SARS-CoV-2 and hyperphosphatemia.

## Introduction

Severe acute respiratory syndrome coronavirus 2 (SARS-CoV-2) has infected over 12 million people worldwide with over 500,000 deaths. It has led to major societal, economical as well as health implications worldwide. It has been shown to severely impact the respiratory, cardiovascular, neurologic, and renal organ systems [1]. To the best of our knowledge, no study has highlighted the impact this novel virus has on the parathyroid gland so far. We present a case of a 46-year-old Hispanic male with no significant past medical history who presented to the ED with complaints of worsening shortness of breath of one-week duration. The patient was found to be coronavirus disease 2019 (COVID-19)-positive, resulting in acute hypoxic respiratory failure. During the second half of his hospital stay, the patient was found to have hypoparathyroidism as well as elevated phosphate levels. Differentials for the aforementioned disorders were ruled out and the only deducible explanation was heightened inflammatory response caused by the novel virus.

## Case presentation

**Figure 1:**
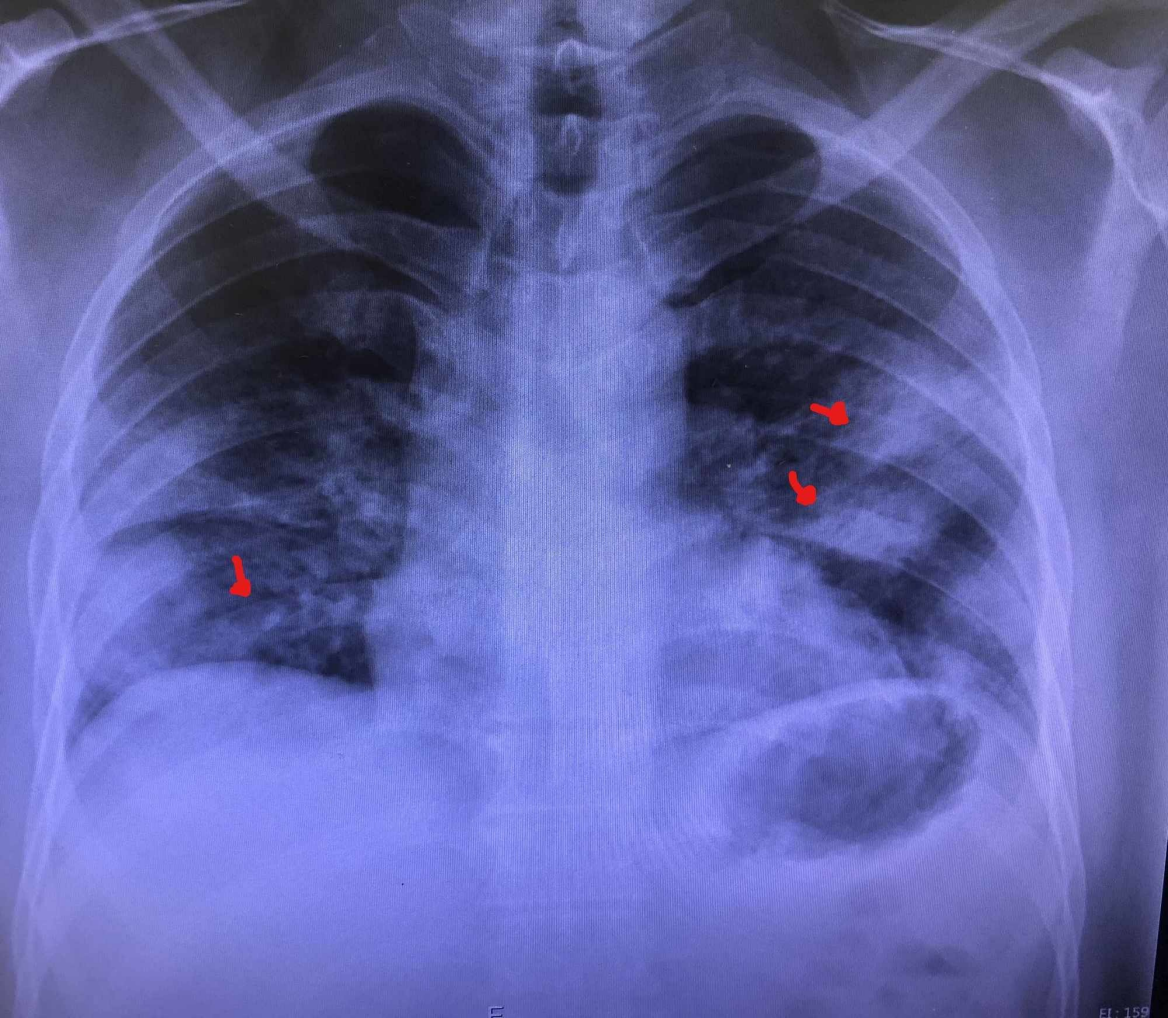
PA chest X-ray showing multifocal pneumonia (arrows) PA: posteroanterior

**Figure 2:**
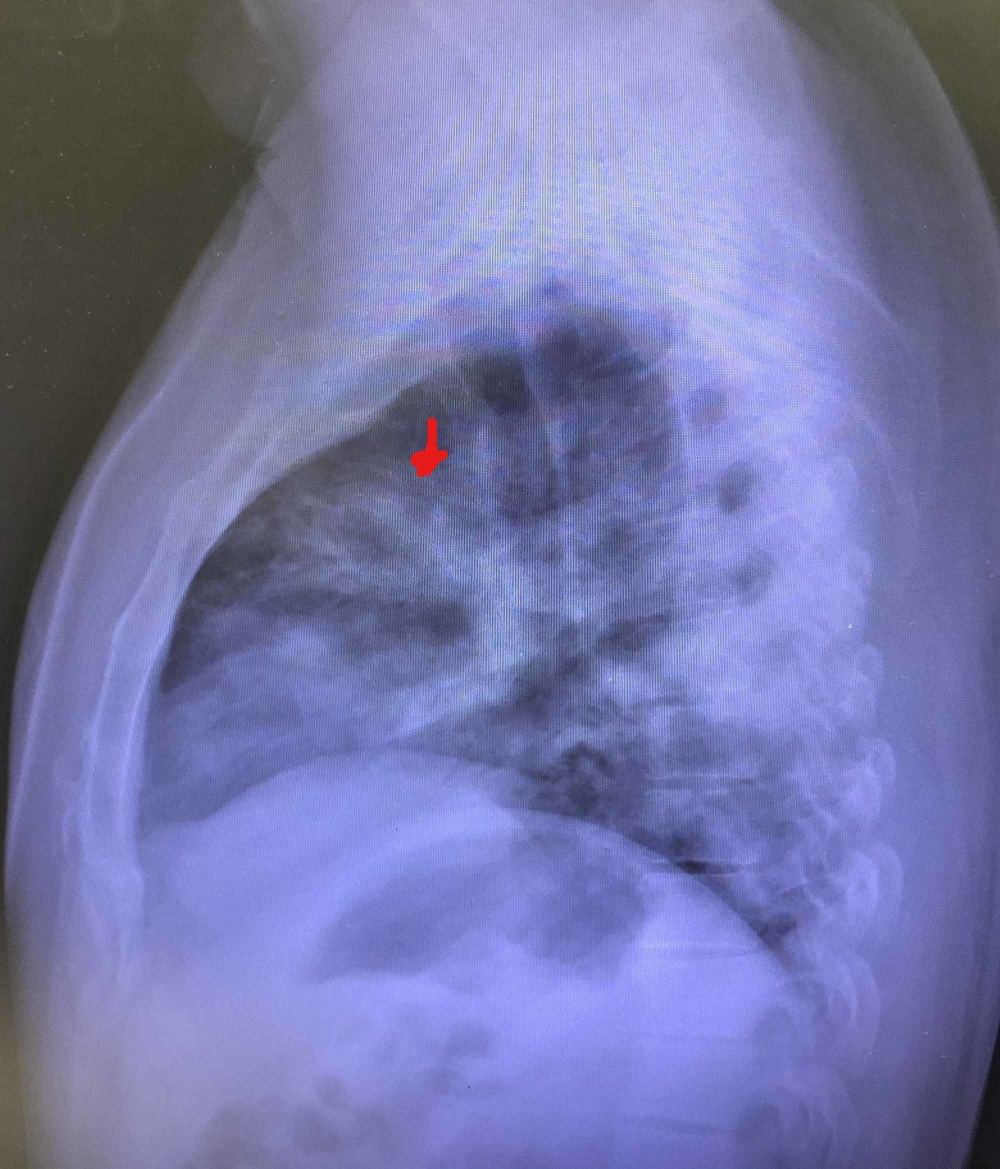
Lateral chest X-ray showing multifocal pneumonia (arrow)

Our patient was a 46-year-old Hispanic male with no significant past medical history who presented to the ED with complaints of worsening shortness of breath of one-week duration. The patient also endorsed fever, chills, body aches, and dry cough. Vital signs on presentation were as follows: temperature: 99.4 F, blood pressure (BP): 123/84 mmHg, and respiratory rate (RR) of 25 breaths/minute with an O_2_ saturation of 90% on room air. Physical examination was unremarkable apart from decreased breath sounds bilaterally. Labs were significant for elevated aspartate aminotransferase (AST) at 67 u/l (normal range: 15-41 u/l) and alanine aminotransferase (ALT) at 84 u/l (normal range: 17-63 u/l). Other lab findings included alkaline phosphatase (ALP) of 225 u/L (normal range: 38-126 u/l), sodium (Na) of 134 mmol/l (normal range: 136-144 mmol/l), potassium (K) of 3.9 mmol/l (normal range: 3.6-5.1 mmol/l). A posteroanterior (PA) and lateral chest X-ray demonstrated multifocal pneumonia (Figures 1, 2). The patient received hydroxychloroquine and azithromycin in the ED. He was initially placed on a 4L nasal cannula with oxygen saturation improvement to 93%. The patient’s saturation decreased to 70% and was placed on non-rebreather with an oxygen saturation of 88%. Hence, he was placed on continuous positive airway pressure (CPAP) therapy with an improvement of oxygen saturation to 96%. He was admitted to the medical floors for oxygen support and further evaluation.

Of note, the patient tested positive with polymerase chain reaction (PCR) nasal swab for SARS-CoV-2. He was continued on azithromycin and hydroxychloroquine on the medical floor. His respiratory status deteriorated within one week of hospitalization, and he was upgraded to the ICU and subsequently intubated for respiratory support and airway protection. His ICU course was complicated by a septic shock from an unknown source initially started on broad-spectrum coverage, meropenem for seven days. The patient continued spiking intermittent fevers and repeat sepsis workup revealed Pseudomonas urinary tract infection, which was treated with ciprofloxacin. The patient was also noted to have marked elevation of D-dimer levels at >5,000. The venous duplex was unremarkable with a transthoracic echocardiogram showing right heart strain. The patient at that point was unstable for CT angiography (CTA) of the chest; hence, he was empirically started on therapeutic anticoagulation. Hematology was consulted given hospital protocol at that time for tocilizumab and convalescent plasma consideration. The patient received tocilizumab twice as well as convalescent plasma. Of note, by the time remdesivir was available at our hospital, the patient had already been retested negative for the novel virus.

Hospital course was further complicated by transient thrombocytopenia for which hematology service was already on board, and anticoagulation was suspended. He received a total of six units of platelets with significant improvement in his platelet count. The patient at that time became stable, and CTA of the chest showed no pulmonary embolism. Unfortunately, hospital stay was complicated by yet another infection, Pseudomonas pneumonia, for which he received meropenem and ciprofloxacin. Eventually, the patient underwent a tracheostomy after one month of intubation and was then downgraded to the medical floors for further management. As he remained on a tracheostomy-to-ventilator initially, he started spiking intermittent fevers and pan cultures were performed, and tracheostomy culture grew carbapenem-resistant Enterobacteriaceae (CRE)-Klebsiella pneumonia sensitive to amikacin. The infectious disease unit was consulted and the patient was started on amikacin and ceftazidime-avibactam for 10 days. The patient also developed diarrhea that coincided with the spiking of fevers and Clostridioides difficile toxin was detected, and he was treated with oral vancomycin. Post-course of antibiotics, the patient stopped spiking fevers with a resolution of diarrhea. He passed weaning and was placed on a trach collar with an improvement in oxygen requirements.

Once the patient was downgraded to medical floors, he was noted to have unexplained hyperphosphatemia during the second month of hospitalization, with phosphorus levels elevated to 6.9 mg/dl (normal range: 2.4-4.7 mg/dl). Blood work during that timeframe revealed the following: Na: 138 mmol/l, K: 4.4 mmol/l, chloride (Cl): 96 mmol/l (normal range: 101-111 mmol/l), bicarbonate: 30 mmol/l (normal range: 22-32 mmol/l), blood urea nitrogen (BUN): 8 mg/dl (normal range: 8-20 mg/dl), creatinine: 0.223 mg/dl (normal range: 0.7-1.2 mg/dl), glucose: 99 mg/dl (normal range: 74-118 mg/dl), phosphorus: 5.8 mg/dl with albumin of 2.9 g/dl, and calcium level was normal at 9.2 mg/dl. Complete blood count (CBC) revealed hemoglobin of 10.2 mg/dl, hematocrit of 32%, white blood cell count of 11.1 k/ul, and platelets of 359 K. Parathyroid hormone (PTH) levels of 8 pg/ml were observed and repeat levels the next day for confirmation revealed a level of 10 pg/ml (normal range: 12-88 pg/ml). Vitamin D level obtained was low (7 ng/dl) (normal range: 30-100 ng/dl), magnesium level was 1.9 mg/dl (normal range: 1.8-2.5 mg/dl), and creatine phosphokinase (CPK) level was 64 u/l (normal range: 38-174 u/l). As vitamin D levels were low, the patient was started on 50,000 units of ergocalciferol once a week. Endocrinology and nephrology consultations were obtained. Parathyroid hormone-related peptide (PTHrP) levels were obtained to rule out paraneoplastic syndrome as a cause of decreased PTH levels. However, it was found to be low at 10. He was started on calcium acetate 1,334 mg three times a day for hyperphosphatemia. Phosphorus levels remained elevated, and sevelamer was added on discharge after he was weaned off oxygen and cleared by physical therapy.

One month following discharge, repeat labs were obtained showing calcium of 9.5 mg/dl, creatinine of 0.75 mg/dl, albumin of 4.1 g/dl, K of 4.5 mmol/l, phosphorus of 5.4 mg/dl, and PTH of 9 pg/dl. A summary of the patient’s labs can be found in Table 1.

**Table 1:** Summary of pertinent laboratory findings over the course of hospitalization Ca: calcium; PTH: parathyroid hormone

Variable	First day of hospitalization	One week after hospitalization/first day of intubation/ICU transfer	Five weeks after hospitalization/day of downgrade to medical floor	Two months after hospitalization	At the time of discharge	One month after discharge
Ca (mg/dl)	8	7.6	7.9	9.2	9.7	9.5
Phosphorus (mg/dl)	4.2	4	5.1	5.8	5	5.4
Albumin (g/dl)	2.7	1.8	2.4	2.9	3.6	4.1
Creatinine (mg/dl)	0.6	1.4	0.2	0.2	0.37	0.75
PTH (pg/dl)				8	16	9

## Discussion

Cases of viral pneumonia that were first identified in China’s Wuhan City was formally attributed to COVID-19 on March 11, 2020 [1]. Since then, there have been tremendous effects worldwide as the virus spread to more than 200 countries [2]. According to Johns Hopkins University data, there have been a total of 14,735,331 confirmed cases and 610,586 global deaths as of July 2020 [3]. The United States alone has seen a total of 140,909 COVID-19-related deaths [3]. While COVID-19 mainly causes lung injury and severe hypoxic respiratory failure, many studies have reported extrapulmonary manifestations as well. Some of the recent data have suggested neurological, cardiac, gastrointestinal, ocular, renal, and cutaneous manifestations of COVID-19 [1,2,4]. However, there is no indication in the current data showing a link between SARS-CoV-2 and hypoparathyroidism. 

Two of the most common etiologies of hypoparathyroidism are either postsurgical following a thyroidectomy, parathyroidectomy, or radical neck dissection, all of which were ruled out in our patient, or autoimmune polyglandular syndromes [5]. The latter is often associated either with cutaneous manifestations such as vitiligo or chronic mucocutaneous candidiasis, or with primary adrenal insufficiency. In our case, the patient did not show any of these manifestations. Other possible etiologies include radiation-induced destruction of the parathyroid gland, excessive accumulation of iron, and copper or infiltration of the parathyroid gland by granulomatous diseases or metastatic disease [5]. Many of these etiologies were ruled out in our patient.

Complex genetic defects and several gene mutations have been documented to cause abnormal growth of the gland leading to inappropriate biosynthesis and secretion of PTH. One common example is mutations in the calcium-sensing receptor (CaSR), which follows an autosomal dominant pattern [5]. A key feature of these genetic disorders is hypocalcemia. It was unlikely that the patient carried these genetic defects given his age and the normal calcium levels. Additionally, PTHrp levels were normal, effectively excluding an underlying malignancy that could lead to low PTH levels. We were leaning towards hypoparathyroidism causing hyperphosphatemia given that the kidney function was intact and the patient had a daily intake of only 1,200 mg of phosphorous.

To date, there have been some reports linking SARS-CoV-2 to widespread tissue injury; however, there has been no report on the effect of the parathyroid gland [1]. Because of the widespread organ injury and damage, COVID-19 may likely contribute to inflammation of the parathyroid gland. Our findings of hypoparathyroidism and elevated serum phosphate levels have implications when it comes to the management of patients with SARS-CoV-2. Further studies are necessary to elaborate and to confirm the causative relationship between SARS-CoV-2 and the reported hyperphosphatemia manifestations of COVID-19 and to determine whether or not it is necessary to monitor the levels of PTH, serum phosphorus, and serum calcium in affected patients.

## Conclusions

Hypoparathyroidism is usually associated with genetic or postsurgical factors. Other causes include autoimmune and infiltrative processes. Our patient had a prolonged course of hospitalization, which could have predisposed him to multiple electrolyte abnormalities. However, he had persistent hyperphosphatemia and low PTH levels throughout his hospital course and after discharge. However, it is unknown whether this is related to COVID-19 infection.
